# Modern Gynecology

**Published:** 1893-06

**Authors:** 


Modern Gynecology. A Treatise on Diseases of Women. Comprising the results of the latest investigations and treatment in this branch of Medical Science. By Charles H. Bushong, M. D. Illustrated. New York: E. B. Treat, 5 Cooper Union. Price, $2.75.

The effort of this book, the author states, "is to place before the physician a clear, common-sense statement of the symptoms of the various diseases of the female sexual organs; to indicate in detail the methods of treatment that can be applied by him, and also to indicate in brief the methods requiring the aid of a specially trained consultant of larger experience. The illustrations have been made, some from photographs taken especially for it and others drawn by an artist under the supervision of the author. It is intended for a hand-book for the general practitioner, and is designed to fill a place in progressive medicine. The book is clearly printed and on excellent paper, and will be of use to the profession. The treatment will not be indorsed by homœopathic physicians.

